# Inferred networks, machine learning, and health data

**DOI:** 10.1371/journal.pone.0280910

**Published:** 2023-01-23

**Authors:** John Matta, Virender Singh, Trevor Auten, Prashant Sanjel

**Affiliations:** Computer Science Department, Southern Illinois University Edwardsville, Edwardsville, Illinois, United States of America; Universitá degli Studi di Milano, ITALY

## Abstract

This paper presents a network science approach to investigate a health information dataset, the Sexual Acquisition and Transmission of HIV Cooperative Agreement Program (SATHCAP), to uncover hidden relationships that can be used to suggest targeted health interventions. From the data, four key target variables are chosen: HIV status, injecting drug use, homelessness, and insurance status. These target variables are converted to a graph format using four separate graph inference techniques: graphical lasso, Meinshausen Bühlmann (MB), k-Nearest Neighbors (kNN), and correlation thresholding (CT). The graphs are then clustered using four clustering methods: Louvain, Leiden, and NBR-Clust with VAT and integrity. Promising clusters are chosen using internal evaluation measures and are visualized and analyzed to identify marker attributes and key relationships. The kNN and CT inference methods are shown to give useful results when combined with NBR-Clust clustering. Examples of cluster analysis indicate that the methodology produces results that will be relevant to the public health community.

## Introduction

The social determinants of health [1] are the community, environmental, and societal causes of unwellness, illness, and even death among population groups with uneven access to resources such as gainful employment, insurance, healthcare, transportation, and education. Due to online medical records and advances in survey technology, there is an abundance of health information available concerning both the general public and hidden, or hard-to-find groups, such as injecting drug users (IDUs), people with HIV, and men who have sex with men (MSM). Although the problems of hidden groups are suffered disproportionately by those groups, they often extend to all parts of society, and can be lessened considerably by appropriate public health interventions. The key to the success of these interventions is understanding the data and its implications.

In this study we use data on hidden and hard-to-find groups obtained from the Sexual Acquisition and Transmission of HIV Cooperative Agreement Program (SATHCAP) [2], a multi-site survey taken in the United States in 2006-2008. This dataset is available to researchers upon application to the Inter-university Consortium for Political and Social Research (ICPSR). The survey was conducted via respondent-driven sampling (RDS) [3], a snowball sampling technique that allows a focus on HIV and its transmission within and outside the MSM community, and within and outside the community of IDUs. SATHCAP attempted to examine features that determine the speed, extent, and path of the spread of HIV. Many of these potential features are social determinants of health; in particular, the nature of partnerships and sexual relationships, methods of drug and alcohol use, availability of medical care and insurance, and living and transportation conditions.

Machine learning is an important and relevant tool for understanding medical and health datasets. To analyze the SATHCAP data, we employ a methodology that first converts it to a graph, or network, representation. This process is called graph inference. We make use of 4 different graph inference methods: k-Nearest Neighbors (kNN) [4], graphical lasso (glasso) [5], Meinshausen and Bühlmann neighborhood selection (MB) [6], and correlation thresholding (CT) [7, 8]. The kNN inference method uses geometric distances to determine the graph’s edges. Glasso and MB use a covariance matrix and create a graph based on applying variations of lasso regression to it. CT chooses edges based on how values in a correlation matrix relate to a specified threshold. There are many reasons that it is desirable to convert data to a network model via graph inference. First is that “correlation networks can be used to find clusters (modules) of interconnected nodes,” [9] which is the primary purpose of this paper. Other goals are to define significant modules, as well as to use network science tools and metrics, such as centrality and closeness, to find significant nodes. Additionally, graph inference is a good solution for large data in that it allows potentially complex relationships to be expressed simply, as the graph’s binary relationships of nodes and edges. This facilitates finding previously unseen connections between features [10, 11]. Graph inference also allows the ability to use the wide range of tools, algorithms, and software that are available from the network science community. This includes analysis and interpretation of results by visualization. The SATHCAP data are robust and lend themselves to many types of analysis. For example, the data can be subjected to standard statistical analysis [12, 13], the referral chains can be interpreted as complex networks [14], and the survey design itself can be used to learn more about RDS [15]. SATHCAP is used here because of previous successful application to a similar dataset [16]. Analysis of this new dataset is one of the novelties of the current work.

Clustering, or community detection, can be used to find similarities among groups identified by shared attributes. In this paper, we use clustering to differentiate health information based on social determinants such as drug use, homelessness, and access to insurance, as well as to determine attributes leading to HIV positive diagnosis. We cluster graphs inferred from the SATHCAP data with a goal of uncovering hidden relationships for use in creating public health interventions. From a machine learning perspective, our approach is completely unsupervised. Graphs are created and clustered, and the most promising clusterings are chosen based on a suite of evaluation measures. We visualize and analyze the clusters and their relationships to each other, and finally comment on the public health implications of the results.

This paper is an extension of *Inferred Networks and the Social Determinants of Health* [16]. The previous paper was a proof of concept for a methodology of graph inference and analysis that showed some success with the glasso and MB inference methods, as well as with the NBR-Clust clustering framework [17]. The current paper is an attempt to show the generalization of those results by using a different, though related dataset, and by showing results for an additional graph inference method. We are especially interested in whether additional graph inference methods can produce good results. Here we test the methodology using a much larger dataset (643 subjects in the previous dataset vs 4685 here). The previous paper analyzed results based on 3 target variables: self-esteem, HIV, and alcohol use. Here we also do HIV analysis for comparison and expand the paper by testing 3 additional target features: injecting drug use, homelessness, and insurance status. We continue to use the glasso and MB inference methods that were successful in the previous study. We compare results with graphs inferred using the classical kNN method and expand the work by testing a new method, correlation thresholding (CT), that is popular in the biological community [9] because of speed and positive results. We find that CT gives promising results with the SATHCAP data, as does kNN. As in the previous paper, we use 4 clustering methods and 5 cluster evaluation measures. We find that the NBR-Clust framework is able to discover interesting and useful clusters, however the low-complexity Leiden and Louvain clustering methods also work well.

## Related work

The need to examine large datasets, particularly with biological and DNA data, has led to the creation of a variety of graph inference techniques. The four graph inference methods employed in this paper have all seen use with medical data applications. The MB inference method has been used in discovering causal relationships between gene regulatory networks [18] and in modeling microbial ecological networks [19]. Glasso [5] has been used extensively in the medical field, for diverse applications such as estimating the network structure of lung cancer gene sets [20], diagnosing endometriosis in teenage girls [21], and investigating the relationship between anxiety and depression symptoms [22]. Glasso has also been used with network analysis to identify relationships between suicidal individuals and suicide attempts [23]. KNN has seen wide use as a network inference method. It has been used with community detection to investigate the effects of mechanical stress on knee joints [24], to analyze the phenotypic and functional diversity in cells of probands with acute myeloid leukemia [25], and to determine conditions leading to death after stroke [26]. CT inference is very similar to weighted gene co-expression network analysis [9], which has been used widely in the biological community. Examples include finding genetic markers for breast cancer [27] and powdery mildew disease in grapes [28].

There exist a large number of network analysis tools and clustering techniques. Clustering and community detection have seen success in the past with medical and biological data. Examples include clustering patent phenotype data in correlation to age and sex [29], clustering based on data obtained via radiothermometric examination which discovered two categories of patients with tumors from oncological diseases [30], and clustering based on various dimensions of multi-morbidity diseases [31]. In Bryant et al., the authors create a network of PTSD symptoms which are used to determine associations between them [32]. In their specific approach, two symptom networks are compared based on patient assessment, with one assessment taking place during hospital admission and the second taking place 12 months later. As in the current study, they utilize glasso as a graph inference technique to determine the correlation between the nodes (symptoms) of their networks. Our study differs in that it clusters probands as opposed to symptoms.

Network analysis and community detection in use with questionnaires can be seen in Puga et al., where the authors identify phenotype communities for tinnitus patients [33]. They form a multi-layered network from multiple questionnaires, with each questionnaire representing a layer in the network. They look at the extent to which tinnitus patients can be represented with this type of network and discern how layers affect the prediction of treatment outcomes. Their community detection is done on each layer individually using the Leiden algorithm, which is one of the four algorithms used in our study. For future work, the authors suggests that evaluating alternative community detection algorithms could be valuable. Although our approach is very different, we attempt to study the differences in results from varying clustering algorithms.

Other machine learning techniques have seen use in regards to both network and clustering analysis. In Hu et al., complex network and machine learning methods are used in the analysis of insomnia symptoms and symptom relationships [34]. The authors use node embedding on their network and construct a symptom vocabulary in a vector format. They then divide the symptoms into communities using spectral clustering and identify core symptoms that always appear in the diagnosis of insomnia. Spectral clustering is a classical machine learning technique, although its high time complexity limits its use.

In each of these works, the datasets are not only clustered, but the clusters are examined to gain insight into the hidden relationships involved. This paper applies machine learning techniques to the SATHCAP dataset in a way that considers the practicality, complexity, and resulting public health relevance of the approaches.

## Methods

**Fig 1:**
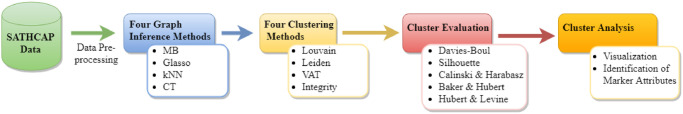
Overall workflow for inferring SATHCAP data.

In this work, feature selection, graph inference methods, clustering, and cluster evaluation are used to analyze the SATHCAP dataset. Specifically, our dataset is converted into multiple network representations and clustered, with the best of these resulting clusterings being analysed for both similar and distinctive features. This work was approved by the Southern Illinois University Edwardsville IRB, and written consent was obtained. The framework of this process is visualized in Fig 1, and each step is described below.

### Data preprocessing

#### Respondent-driven sampling and the SATHCAP dataset

Our study makes use of SATHCAP, a dataset related to the transmission of HIV via sexual activity and drug use, collected using RDS [3]. SATHCAP recruitment started with seed nodes chosen by researchers. As the survey continued, each participant was given coupons that could be used to recruit others into the study, provided that they were members of a target group likely to engage in HIV-transmitting behaviors. It has been shown that, if certain conditions are met, and enough recruitment waves occur, RDS data are statistically independent of the initial seed nodes. In particular, it is stated that equilibrium (i.e., when over-sampling or under-sampling due to recruiting level out) is reached with a tolerance of 2% within approximately six recruitment waves [3]. As an example, in the initial SATHCAP surveying of Los Angeles, 12 seeds led to 576 participants, which gives a smallest possible chain length (if all participants recruit 3 others) of *log*_3_(576) = 5.785, approximately meeting this requirement [35]. Because of this chain length, we assume that the SATHCAP data are a statistically independent sample. Some of the other conditions required for a successful RDS survey are that respondents are likely to know other people in the hard-to-reach population (and are able to count how many they know), that respondents recruit others uniformly at random from their network of friends, and that a sample’s recruitment probability has an inverse relationship with the size of the sample’s friendship base, implying that subjects with more social connections are recruited earlier in the process.

The data collection involved three US cities: Chicago, IL; Los Angeles, CA; and Raleigh, NC. Across all sites, the survey included 4,685 participants who were asked nearly 1500 questions. The answers to the questions are referred to as *features, attributes, or variables*. For example, one variable is *transportation status*, which refers to the question “What is your primary form of transportation?” Possible values for this variable are *car, bus*, and *walking*. The overall purpose of SATHCAP is to research behavioral, biological, and environmental determinants in the spread of HIV and other STDs among both drug users and non drug-users [36]. For this reason, most of these questions were related to the participant’s sexual and drug habits, although there are many other questions such as the participant’s education level, alcohol use, and living situation. The large number of variables collected for this dataset make it applicable for studying hidden relationships involving a variety of public health issues.

#### Data curation

The first step in data curation was to remove any variables that would interfere with feature selection. This includes metadata, such as participants’ coupon keys, along with other variables, such the location where the participant was interviewed. It should be noted that, because of the large number of questions on the SATHCAP survey, coupled with their private nature, participants were given the freedom to skip questions. In practical terms, this means there are a large number of features with low participation, with 1352 of the 1493 total features missing 40% to 99% of their values. After removing irrelevant features, continuous variables, and features with a significant amount of missing data, we were left with a total of 48 features. 12 of these 48 features were multi-valued and converted to binary variables using one-hot encoding. As an example of one-hot encoding, participant age is changed from a variable with a wide range of answers to three binary variables; *Age: between 18 and 30*, *Age: between 31 and 45*, and *Age: 46 or greater*. After one-hot encoding, the total number of features increased to 98. The remaining low number of answers marked as skipped or anything other than yes or no were converted to the answer no. As in [37], the features were pruned by applying a pairwise correlation filter using Pearson coefficient. The features with a correlation greater than 80% were discarded. This step was done to reduce the risk of multicollinearity between the independent variables during the logistic regression feature selection [38] described in the following section.

#### Feature selection

Following the pre-processing, feature selection using logistic regression was run with the purpose of selecting the most relevant of our features with respect to each target variable: HIV status, injecting drug use, homelessness, and insurance status. Logistic regression, which uses a linear model for binary classification, has been shown to perform well on a variety of medical data [37, 39]. The step forward algorithm was used as the wrapper method. Step forward is a greedy algorithm which works by setting the best performing classifier as the base feature with which all other features are combined as a possible set. The features that perform the best in this combination are added to the set, and this process is repeated until the feature set reaches the desired size. ROC area under the curve (AUC) was used as our performance evaluation criteria, and the performance of the algorithm was evaluated on training and test sets for each target feature. The final result of our feature selection process reduced the number of variables from 98 to the best feature sets of size 15, 20, and 64 for each of the four examined attributes.The exact variables and the feature sets they are contained in are shown in Table 1. Logistic regression and the step forward wrapper were implemented using the Scikit-Learn [40] and Mlextend [41] Python packages, respectively.

**Table 1:** A list of all 64 variables used in the features selection along with the best features of size 15 and 20 for each of the four target attributes.

	HIV		Injected Drugs		Homelessness		Insurance	
Variables	15	20	15	20	15	20	15	20
Race: American Indian or Alaska Native		**X**						
Race: Asian or Pacific Islander								
Race: Black / African American			**X**	**X**		**X**		
Race: Other race								**X**
Race: Spanish/Hispanic/Latino	**X**	**X**		**X**				
Race: white				**X**				
Drugs: methamphetamines	**X**	**X**	**X**	**X**	**X**	**X**		
Drugs: sedatives	**X**	**X**	**X**	**X**				
Drugs: crack			**X**	**X**	**X**	**X**		
Drugs: heroin	**X**	**X**	**X**	**X**	**X**	**X**		
Drugs: heroin and cocaine together			**X**	**X**				
Drugs: injected drugs		**X**	**X**	**X**	**X**	**X**		
Drugs: marijuana		**X**	**X**	**X**		**X**		
Drugs: none		**X**	**X**	**X**			**X**	**X**
Drugs: opiates							**X**	**X**
Drugs: other drugs				**X**	**X**	**X**	**X**	**X**
Drugs: powder cocaine								
STD: Chlamydia								**X**
STD: Gonorrhea								
STD: HIV	**X**	**X**	**X**	**X**		**X**	**X**	**X**
STD: Syphilis	**X**	**X**						
Age: 18–30		**X**						
Age: 31–45	**X**	**X**			**X**	**X**		
Age: 46+				**X**			**X**	**X**
First had sex: 0–12								
First had sex: 12–18								
First had sex: 19+								
Drinks in past month: 0–9	**X**	**X**						
Drinks in past month: 10–19								
Drinks in past month: 20+								
First drink: 0–12			**X**	**X**				
First drink: 12–18						**X**		
First drink: 19+							**X**	**X**
First got drunk: 0–12								
First got drunk: 12–18								**X**
First got drunk: 19+								
Have been homeless in past year	**X**	**X**	**X**	**X**	**X**	**X**	**X**	**X**
Have been to prison or jail			**X**	**X**			**X**	**X**
Have partner who has been to prison	**X**	**X**	**X**	**X**	**X**	**X**		
Have health insurance	**X**	**X**			**X**	**X**	**X**	**X**
Difficulty getting health care due to alcohol/drug use	**X**	**X**		**X**	**X**	**X**		
Difficulty getting health care due to culture or language								
Difficulty getting health care due to HIV status	**X**	**X**		**X**			**X**	**X**
Difficulty getting health care due to national origin								
Difficulty getting health care due to race/ethnicity								
Difficulty getting health care due to sexual orientation					**X**	**X**	**X**	**X**
Difficulty getting health care due to social / economic status					**X**	**X**	**X**	**X**
Difficulty getting health care for any other reason	**X**	**X**					**X**	**X**
Participated in any self help groups	**X**	**X**			**X**	**X**		
Been in formal treatment program for drug or alcohol			**X**	**X**				
Month income: $0–$500					**X**	**X**	**X**	**X**
Month income: $501–$1000	**X**	**X**						
Month income: $1001–$1500								**X**
Month income: $1501–$2000								
Month income: $2001–$2500								
Month income: $2501–$3000								**X**
Month income: $3001–$4000								
Month income: $4001–$5000								
Month income: $5001+								
Sexual partners in 6 months, 0–1					**X**	**X**		
Sexual partners in 6 months: 2- 5					**X**	**X**		
Sexual partners in 6 months: 6–10							**X**	**X**
Sexual partners in 6 months: 10+								
Received money or goods for sex						**X**	**X**	**X**

### Graph inference

A total of four inference methods were used to convert the SATHCAP data into graphical formats. The first inference method is the classic k-Nearest Neighbors (kNN). In kNN, each node is considered to be a vector of features, and the distance (in our case, Euclidean) is calculated between each pair of node vectors. For each node, edges are then placed between the node and its *k* nearest neighbors. The edges are undirected, although a nearest-neighbor relationship may not be symmetrical. This implies that a kNN graph has an average degree close to, but not necessarily exactly equal to, *k*. Because of evidence in favor of use of minimum connectivity graphs [42], all kNN graphs were constructed using the minimum *k* that resulted in connectivity. Minimum connectivity graphs can have smaller communities of dense edge groups along with few inter-cluster links. Depending on the clustering technique, this structure can be expected to produce a large number of clusters. Given that the goal is to find clusters that illuminate the importance of a small number of variables, this approach serves our purposes well.

The next two inference methods use a covariance matrix and create a graph based on variations of lasso regression. A great deal of work has been done on finding efficient ways to perform this lasso regression. Meinshausen and Bühlmann [6] proposed a probabilistic neighborhood selection method, which estimates a sparse graph by fitting a lasso regression model to each variable, estimating the conditional independence restrictions separately for each node in the graph. The results for these nodes are then combined, creating the structure of the graph. This neighborhood selection technique is proved to have a lower, approximately quadratic, computational complexity than standard covariance selection in the case of sparse high-dimensional graphs [6]. The resulting graph inference method is called MB and can be used with large datasets.

The third graph inference method is the graphical lasso (glasso), proposed by Friedman et al. [5]. Glasso is a simple algorithm for quick estimation of a sparse inverse covariance matrix using a lasso penalty. It works by fitting a modified lasso regression to each variable, solving the lasso problem for each variable using coordinate descent [43]. Both glasso and MB inference methods were chosen because of previous empirical evidence showing good results for methods that utilize lasso regression [16].

The final inference method used is correlation thresholding graph estimation (CT) [7]. CT creates a graph by applying a threshold to values in a correlation matrix. An edge is placed depending on whether the correlation value is greater or less than the value of the threshold. This is similar to constructing a geometric graph, where nodes within a certain distance are linked, and the correlation value serves as a distance measure. This method is desirable because of its comparatively low time complexity.

The MB, glasso, and CT networks were created using the High-Dimensional Undirected Graph Estimation (huge) R package [7]. The kNN networks were created using the CCCD R package [44]. All graphs were created with the minimum number of edges possible, as all of these inference methods assume a sparse network. Using these graphs with clustering methods based on density of edges may lead to a large number of clusters. It is possible that fewer clusters could give better results. One way to obtain fewer clusters would be to join adjacent clusters until a desired number of clusters is obtained. We have avoided this, as the optimal number of clusters is not known, and all clustering methods employed here determine the number of clusters as part of creating a partition.

### Clustering

Four clustering methods are used in our study: Louvain [45], Leiden [46], and the NBR-Clust Framework [17] with VAT [47, 48], and integrity [49]. Louvain is a popular and fast algorithm based on maximizing modularity i.e., the strength of division between clusters. Leiden is faster than and intended to be a direct improvement to Louvain by “converging to a partition in which all subsets of all communities are locally optimal.” Leiden also yields communities that are guaranteed to be connected [46]. The NBR-Clust framework [50] uses graph resilience measures to determine attack sets of nodes which are then removed from the graph to create clusters. Here we have used vertex attack tolerance (VAT) and integrity as resilience measures. Since VAT and integrity produce different attack sets, they may result in different clusterings. With VAT, the attack set S ⊂ V is selected to minimize the equation

$$\begin{array}{c} VAT\left(G\right)=\underset{S\subset V}{\text{min}}\{\frac{\left|S\right|}{|V-S-C_{max}\left(V-S\right)|+1}\}, \end{array}$$ (1)

and integrity is selected to minimize the equation

$$\begin{array}{c} I\left(G\right)=\underset{S\subset V}{\text{min}}\{\left|S\right|+C_{max}\left(V-S\right)\}, \end{array}$$ (2)

where V is the set of vertices, S is the attack set, and *C*_*max*_ is the size of the remaining largest connected component.

While some of the clustering methods used show more promise than others, e.g., Leiden over Louvain, there is merit in utilizing multiple methods as actual results can vary between different datasets and the graphical representation of those datasets. With all methods, the number of clusters obtained is decided as part of the clustering algorithm.

### Cluster evaluation

This paper is an extension of [16], in which the methodology presented in [42] was followed. We continue to employ this methodology, as the purpose of this paper is to examine its use when mining larger, similar medical data sets, with networks inferred using different algorithms. The SATHCAP dataset is good for this examination, as it includes variables specifically for HIV as well as other attributes present in the dataset used in [16].

We quantified the goodness of our clustering results using a total of 5 internal evaluation measures. The purpose of clustering evaluation is to measure and quantify desirable clustering properties, such as maximal separation between clusters combined with minimal separation within a cluster. The evaluation methods used are as follows: Davies-Bouldin, Silhouette, Calinski-Harabasz, Baker-Hubert, and Hubert-Levine. All clustering evaluation was done using the ClusterSim R package [51]. With Davies-Bouldin and Hubert-Levine, a lower score indicates a better clustering. A higher score indicates a better clustering for the other three methods. Each clustering result was given a number of points, with clusterings ranked higher based on the number of best scores received for each evaluation measurement. An example of clustering scores is shown in Table 2. These are scores for the CT graph for HIV clustered using Louvain and NBR-Clust with integrity. As highlighted in yellow, the CT graph created with 64 attributes and clustered by integrity scores best on Davies-Bouldin, Silhouette and Hubert-Levine. It receives 3 points. The Louvain graph created with 20 attributes scores best with Calinski-Harabas and Baker-Hubert, receiving 2 points. In this case, with three of the five best scores, the CT-integrity-64 is the HIV clustering chosen for further analysis. Scores were calculated for each target across the four clustering algorithms and the four inference methods. As such, the best scores could be widely distributed, and ties were possible. Ties were broken by directly comparing the scores of the tied clusters and giving a point to whichever had the better of each individual score. The tie was broken in favor of the cluster with the most points.

**Table 2:** Example of evaluation scores and methodology (Actual scores for the CT graphs). Best scores are highlighted in yellow.

Clustering	#Attributes	Davies-Bouldin	Silhouette	Calinski- Harabas	Baker-Hubert	Hubert-Levine
Louvain	15	6.5642	-0.2268	8.3909	0.1874	0.4581
	20	5.5995	0.0407	18.7714	0.3495	0.3985
	64	4.4396	0.0304	18.2146	0.1927	0.3735
Integrity	15	3.9165	-0.2125	2.9952	-0.1096	0.5888
	20	2.8065	-0.1429	5.3985	0.1375	0.5088
	64	1.2588	0.2572	13.8216	0.8112	0.1389

## Results

Using the evaluation methodology presented above, the top clustering result for each target variable was chosen. For HIV, this was the CT graph with 64 variables clustered with NBR-Clust with integrity. For injected drugs, the top result was the kNN 15-variable graph clustered with integrity. The kNN 15-variable graph clustered with Leiden scored best for homelessness, and the kNN 15-variable graph clustered with Louvain scored best for insurance status. We focus on these clusterings for further visualization and analysis. Both here and in [16], the NBR-Clust method produced some of the best results. It is stated in [17] that node-removal clustering methods such as NBR-Clust are effective at identifying noise, creating cleaner clusters than other methods. As medical data are inherently noisy, this may be the reason for NBR-Clust’s good performance. The composition of variables for each cluster for the top clusterings is shown in Table 3. The label and label color for each column corresponds to a cluster in the network’s visualization. The numbers in the table represent the percentages of cluster members that display that variable. In the interest of brevity, we are not able to examine all the clusters produced, although a practitioner conducting a similar study would want to. We have tried to choose clusters that represent the most interesting aspects of the results. This includes clusters that are close in attributes (and close in the graphical representation), as well as disjoint and outlier clusters. We note that many of the clusters chosen are inherently similar, and the intent is to show the characteristics that make these seemingly related populations distinct from one another.

**Table 3:** Percentage of each cluster with a positive response for the listed variable. Clusters are coded by their colors on the corresponding figures.

	HIV		Injected Drugs						Homelessness					Insurance					
Variables / Cluster Number	0	1	17	24	40	42	44	46	3	4	8	11	17	16	26	2	3	20	12
Race: Black / African American	62	3	27	100	100	94	100	100	47	58	85	48	28	75	48	61	80	82	84
Race: White	24	53	53	0	0	6	6	0	40	28	7	37	51	20	37	35	16	13	12
Race: Spanish / Hispanic / Latino	15	78	27	0	4	6	0	0	17	14	11	18	21	9	16	4	4	10	12
Drugs: heroin	32	0	100	100	100	0	94	100	78	97	53	84	85	34	54	17	53	55	45
Drugs: crack	68	6	100	100	96	41	100	82	93	89	87	92	83	68	66	26	82	51	53
Drugs: marijuana	81	50	87	100	96	6	100	94	92	92	73	83	94	64	89	100	85	49	67
Drugs: opiates	25	0	40	79	65	6	81	65	60	52	24	54	68	0	80	100	43	0	0
Drugs: sedatives	19	6	0	100	96	0	100	100	54	53	14	49	62	2	44	35	35	7	2
Drugs: cocaine	58	31	93	88	91	12	94	88	83	84	60	73	91	48	75	87	65	48	37
STD: HIV	100	100	0	13	13	0	6	6	14	6	8	7	6	0	30	0	7	0	0
STD: chlamydia	3	3	0	0	4	0	0	0	7	8	3	0	0	0	1	0	3	0	8
STD: syphilis	15	38	0	8	9	24	13	6	10	5	15	10	2	2	8	9	9	10	2
STD: gonorrhea	0	0	0	0	0	0	0	0	0	0	0	1	0	5	1	9	0	0	4
Been to prison or jail	73	22	53	100	91	65	100	100	85	84	86	88	79	100	96	0	77	100	100
Been to self help group	78	22	73	96	91	82	81	94	90	89	83	90	89	73	76	26	79	80	65
Been to formal treatment program	68	6	93	96	96	88	94	100	88	84	83	84	94	68	73	26	77	82	69
Age: 18–30	8	13	27	0	9	0	6	0	14	19	0	5	28	32	31	74	13	0	22
Age: 31–45	54	66	73	0	83	53	94	0	42	16	100	66	0	68	68	26	35	0	77
Age: 46+	38	22	0	100	9	41	0	100	44	64	0	28	72	0	0	0	53	100	0
Month income: $0–$2000	94	97	93	92	100	94	100	100	98	98	100	100	100	100	100	100	96	89	100
Month income: $2000+	5	0	0	8	0	6	0	0	2	2	0	0	0	0	0	0	4	10	0
First drink: 0–12	32	3	100	0	52	75	0	0	49	48	32	55	45	25	39	26	42	21	22
First drink: 12–18	53	47	0	88	48	18	94	94	46	46	53	40	51	59	58	61	43	63	57
First drink: 19+	10	47	0	4	0	6	0	6	2	4	7	3	4	9	3	9	9	9	6
First got drunk: 0–12	21	6	67	0	48	35	0	0	33	35	20	46	28	11	31	13	31	15	18
First got drunk: 12–18	60	31	27	83	52	59	88	88	60	57	59	49	68	61	62	65	53	61	59
First got drunk: 19+	13	59	7	8	0	6	6	12	3	7	14	4	4	20	7	17	10	15	8
Drinks in past month: 0–9	73	94	60	50	52	41	38	65	57	59	39	54	74	73	78	70	51	76	65
Drinks in past month: 10–19	10	0	0	25	9	12	6	6	7	13	16	10	6	7	6	13	19	17	20
Drinks in past month: 20+	13	0	40	17	39	47	50	29	32	27	37	35	19	14	17	13	25	0	0
Age first had sex: 0–12	34	13	33	17	35	41	44	18	29	21	24	28	21	39	18	13	31	11	28
Age first had sex: 12–18	61	66	53	71	61	53	50	82	65	74	58	64	70	50	75	83	64	76	67
Age first had sex: 19+	10	16	13	8	4	6	6	0	6	5	11	5	9	5	6	4	4	85	4
Sexual partners in 6 months: 0–1	30	19	33	33	39	35	31	35	3	0	0	72	98	46	52	22	14	44	39
Sexual partners in 6 months: 2–5	43	31	40	54	30	47	25	47	44	86	0	0	0	41	42	78	35	46	55
Sexual partners in 6 months: 6–9	9	19	0	8	9	6	19	0	24	3	41	98	21	7	0	0	35	3	0
Sexual partners in 6 months: 10+	9	13	13	4	22	12	13	12	21	9	45	15	0	0	1	0	15	1	0
Received money or goods for sex	24	28	47	33	44	29	31	29	53	27	76	32	11	0	7	0	90	0	0
Reside: Apartment, condo, own house, or dorm	45	44	13	42	30	35	13	35	16	17	23	17	21	21	44	52	28	68	35
Reside: Shelter, boarding house, or halfway house	23	9	33	4	22	0	19	18	44	33	26	27	28	25	16	4	25	0	4
Reside: Squat, abandoned building, or the street	3	0	20	0	0	0	0	6	8	5	3	14	9	0	1	0	2	0	0
Education: High school or GED	36	25	67	21	35	24	44	42	38	37	35	41	36	48	28	48	33	28	51
Education: Graduated from 4 your university	7	6	0	8	4	0	0	0	2	0	2	2	0	2	0	0	6	0	0
Marital status: Single (never married)	64	72	60	38	52	42	81	65	46	52	71	53	45	66	68	65	53	31	73
Work status: Disabled / unable to work	53	41	7	29	44	18	25	24	28	32	22	23	38	34	34	30	53	65	41
Work status: Unemployed	33	34	87	42	52	59	50	71	59	57	72	69	45	50	47	61	36	10	45
Work status: Full time	5	13	0	8	0	0	13	0	5	1	2	2	9	2	4	0	3	1	4

### HIV status

**Fig 2:**
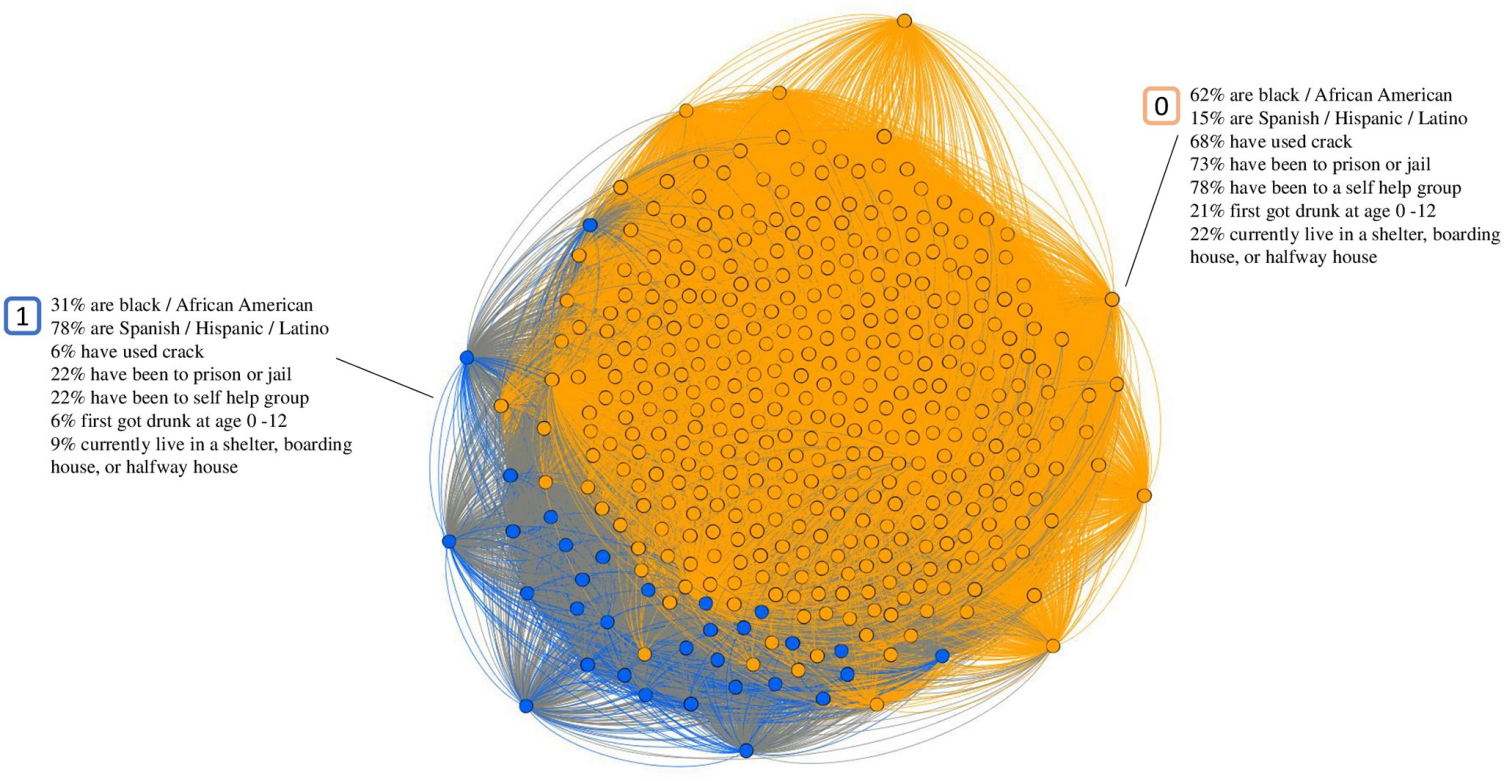
HIV clustering.

Results for individuals who have tested positive for HIV consist of two clusters which are visualized in Fig 2. The majority of members are found in cluster 0, which is 62% black / African American versus 31% in cluster 1. Cluster 1 is notable because of its high percentage of Hispanic members at 78.1%. Members of cluster 0 struggle more with drugs than cluster 1, with 68.3% having used crack, 31.9% having used heroin by itself, and 58.4% having used powder cocaine. This is contrasted with cluster 1, where 6.3% have used crack, 0% have used heroin by itself, and 31.3% have used powder cocaine. 72.8% of cluster 0’s members have been to prison or jail and 77.7% have been to a self help group (such as A.A, N.A., or C.A.). Cluster 0’s members also struggle more with alcohol than cluster 1, with 31.7% having their first drink and 21.5% first getting drunk at age 0–12, as opposed to 3.1% having their first drink and 6.3% first getting drunk at age 0–12 for cluster 1. Alcohol and drug use can be seen affecting the living conditions of members in cluster 0, with 22.5% currently living in a shelter, boarding house, or halfway house. It is interesting that in [16] HIV status is differentiated mainly by the prior occurrence of STDs. The SATHCAP dataset is highly focused on drug use, which is evident in this clustering. However, even here STDs are important, with cluster 1 experiencing syphilis at approximately 3 times the rate of cluster 0.

### Injected drug use

**Fig 3:**
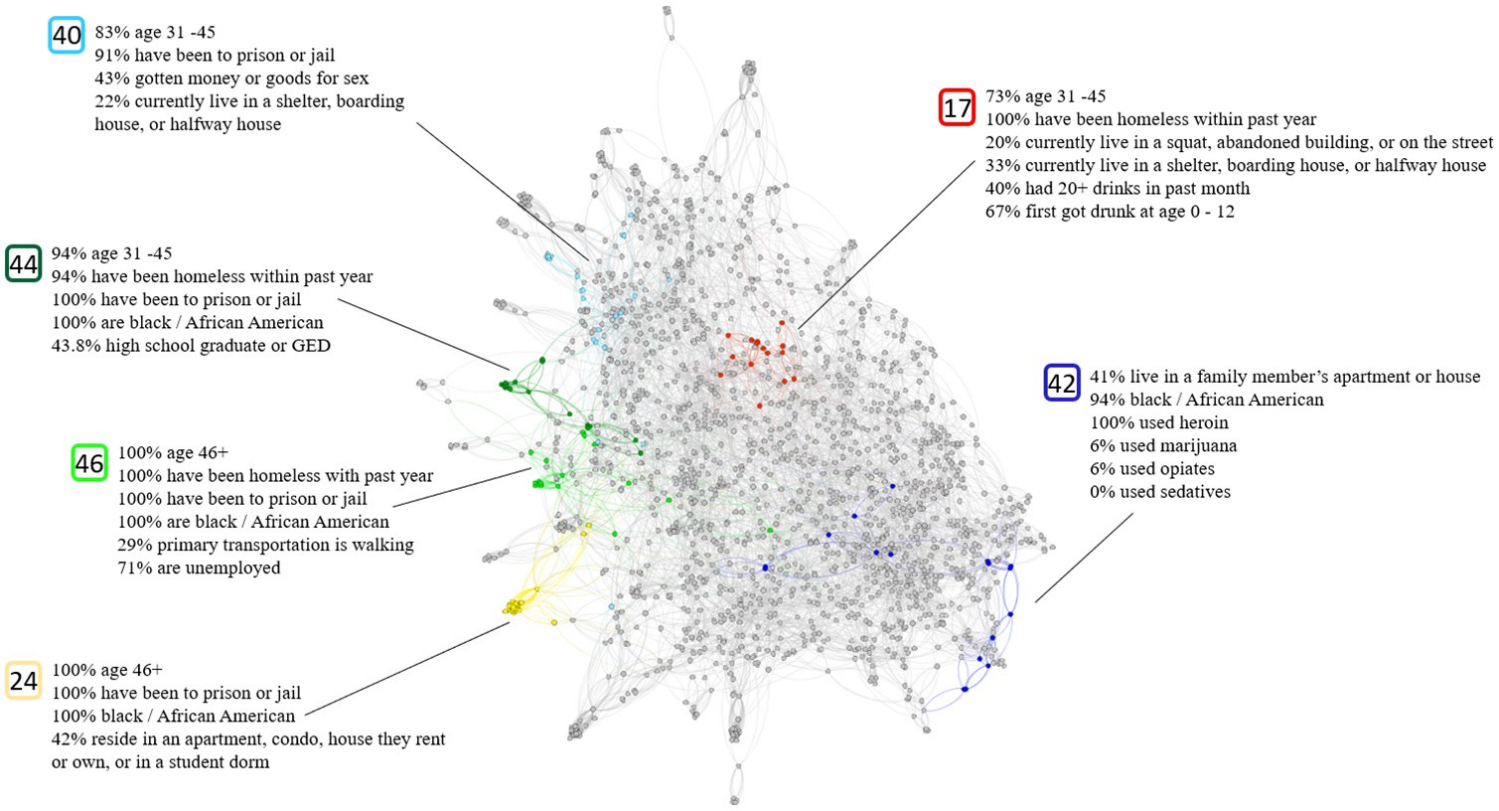
Injected drugs clustering.

Results for individuals who have injected drugs are visualized in Fig 3, with the graph containing 57 total clusters. This large number of clusters is expected, given the low connectivity and overall lack of dense areas in this graph. However, the number of clusters is chosen by the clustering algorithm and not controlled by us. Small clusters of less than 10 nodes are not highlighted or described, both because they often mimic other clusters they are close to, and also to protect the individual identities of participants. Out of the 57 total clusters, 6 are highlighted in the visualization and are detailed in Table 3. It can be seen in the visualization that four of the five clusters are grouped together. We chose these clusters to highlight because we believe it is interesting to see the interplay of different features, and how different features become important as one walks through close clusters.

Cluster 40 is 82.6% middle age and includes prostitution, with 43.4% having gotten money or goods in exchange for sex, along with 91.3% having been to prison or jail. 21.7% are currently living in a shelter, boarding house, or halfway house, which shows that a portion of this cluster’s living conditions are being negatively affected by their lifestyle. Cluster 44 is similar to cluster 40 in that it is heavily middle age (93.7%), and 100% of its members have been to prison or jail. This cluster is made up entirely of black / African American members, and 93.8% of its members have been homeless within the past year. 43.8% have graduated high school or have a GED, and an additional 8.3% have finished 4 years of college or university, making this cluster fairly educated.

Cluster 46 is an older group, with 100% being age 46+. It shares many properties with cluster 44: 100% are black / African American, have been homeless within the past year, and have been to prison or jail. This cluster has a high unemployment rate, with 71% of its members being unemployed. Cluster 24 is another older cluster, with 100% of its members being age 46+. Like clusters 44 and 46, 100% of this cluster’s members are black / African American and have been to prison or jail. 41.7% currently reside in an apartment, condo, house they rent or own, or in a student dormitory, which shows decent living conditions. It is interesting to note the overall closeness of clusters 40, 44, 46, and 24 in the visualization. The clusters are linked by common attributes, such as having been to jail, and having a high proportion of African Americans. The clusters are separated by attributes such as age (though notice the closeness of matching age groups in clusters 40 and 44, as well as the closeness of age in clusters 46 and 24). These clusters are differentiated by living situation, education, and employment status.

Cluster 17 is separated from the previous 4 clusters and includes members who are struggling with both homelessness and alcoholism. 100% have been homeless in the past year, with 20% currently living in a squat, abandoned building, or on the street. 33% currently live in a shelter, boarding house, or halfway house. 66.7% first got drunk at the age of 0–12 and 40% have had 20+ drinks in the past month. Cluster 42 is an outlier. Here we see a very specific group. Specifically, African Americans who have used heroin. Unlike other clusters who have used many different drugs, they have mostly not used other drugs such as marijuana, opiates or sedatives.

Some trends can be seen across all clusters for injected drug users. A large number of injected drug users have been to prison or jail. Many have also been to formal treatment for their drug use.

### Homelessness

**Fig 4:**
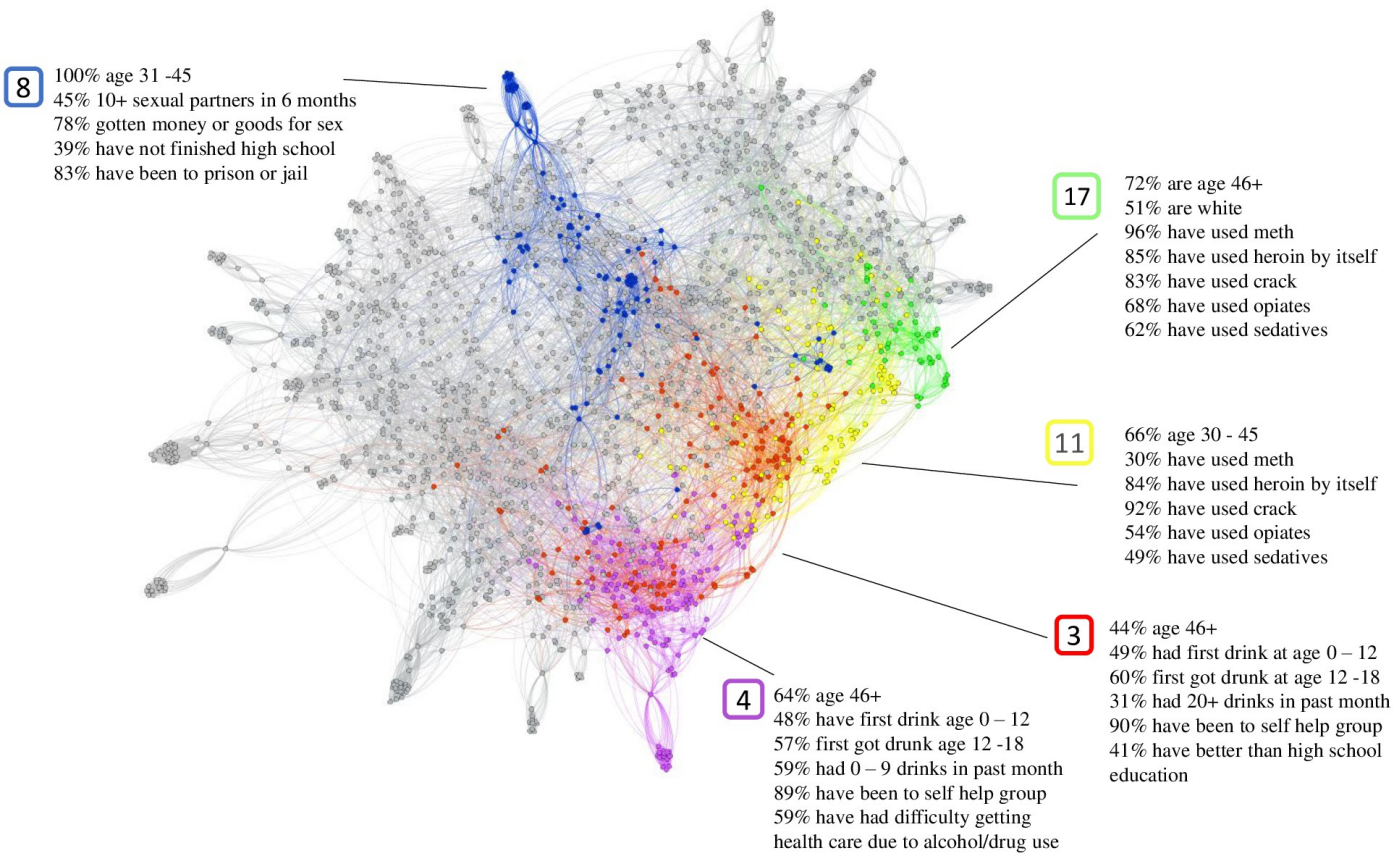
Homelessness clustering.

Results for individuals who have been homeless within the past year are visualized in Fig 4, with the graph containing 23 total clusters. As was the case with injected drugs, we can see outliers as a result of the graph’s low-connectivity. Out of 23 total clusters, 5 are highlighted in the visualization and detailed in Table 3. These 5 clusters were chosen because they are large in size and clearly show differentiating attributes, which for homelessness appear to be sex and prostitution, struggles with alcohol, and struggles with drug use. Cluster 8 is 100% middle age and is distinct for the large amount of sex and prostitution seen. 45% have had 10 or more sexual partners in the past 6 months, and 75.7% have gotten money or goods in exchange for sex. In addition, this cluster is less educated, with 38.7% not finishing high school. A majority (82.9%) have been to prison or jail.

Cluster 3 is dominated by members who have struggled with alcohol. 31.2% have had 20 or more drinks in the past month. 48.9% had their first drink between age 0 to 12 and 60.2% first got drunk between age 12–18. 90.2% have been to self help groups. This cluster is more educated than others, with 41.3% of its members having better than a high school education. Cluster 4 is another cluster of individuals struggling with alcohol. Note the closeness of this cluster to cluster 3 in the visualization, and the overlap between the two clusters. Like cluster 3, this cluster has an older demographic, with 64.4% of its members being age 46 or older. 89.4% have been to self help groups, and 59.1% have had difficulty getting health care due to alcohol or drug use. This cluster differs from cluster 3 in that, with 59.1% having had only 0 to 9 drinks in the past month, many of the members of this cluster are drinking less.

Cluster 11 contains members who have struggled with drug use. 30.4% have used methamphetamine, 83.7% have used heroin by itself, and 92.4% have used crack. In addition, 54.4% have used opiates without a prescription and 48.9% have used sedatives without a prescription. High drug use can also be seen in cluster 17, with 95.8% having used methamphetamine, 85.1% having used heroin by itself, and 82.9% having used crack. 68% have used opiates without a prescription and 61.7% have used sedatives without a prescription. While this cluster shares many attributes with cluster 11, it is differentiated in that it is predominantly older, white members, with 51.1% being white and 72.3% being 46 or more years old.

Homelessness has some common trends seen across all 23 clusters. Like the previous injected drug use clustering, a large number of homeless individuals have been to prison or jail in the past and to self help groups or formal treatment programs for drug or alcohol use. In addition, many are unemployed, but not disabled or unable to work.

### Health insurance

**Fig 5:**
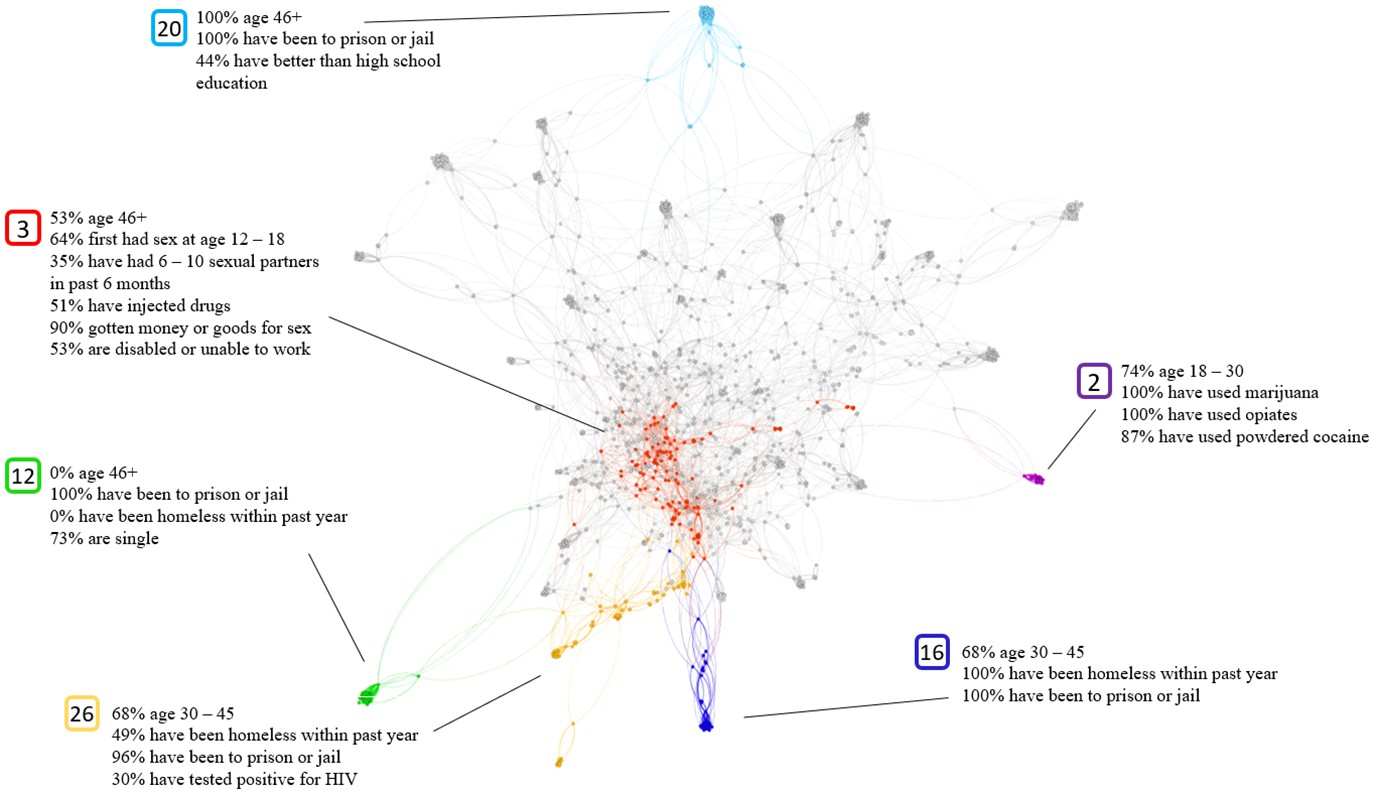
Insurance clustering.

Results for individuals with health insurance of any kind are visualized in Fig 5, with the graph containing 31 total clusters. Here, the outlying clusters resulting from the low-connectivity are more apparent than the previous graphs, with many clusters being isolated in the visualization. Out of the 31 total clusters, 6 are highlighted. The visualization shows that cluster 3 dominates the center of the graph, and most other clusters are outliers. Cluster 3 is a cluster of older members who have had sex at a young age. 34.7% have had 6–10 sexual partners in the past month, and 89.8% have had sex in exchange for money or goods. 53.4% are disabled or unable to work.

The remaining clusters are visualized as outliers. Interestingly, the remaining clusters do not continue the themes of sex at young age, prostitution and inability to work that are prominent in cluster 3. Cluster 2 is a young cluster that struggles with drug use. 74% of its members are age 18–30. 100% have use marijuana, 100% have used opiates without a prescription, and 87% have used powdered cocaine. Cluster 12 is notable mainly because none of its members are older, and none have been recently homeless. Cluster 16 is just the opposite, with more middle-age members, all of whom have recently been homeless. Cluster 20 is an older cluster, with 100% of its members being age 46+. 100% have been to prison or jail, and 44% have better than a high school education. Cluster 26 is between and mixes the results of clusters 12 and 16, with 49% homeless within the last year. This cluster is differentiated because of its high rate (30%) of HIV positive members.

## Discussion

We first discuss the clinical and public health significance of the results. For HIV, the presence of 2 clusters has clear implications for outreach efforts. Subjects in the predominantly African American cluster struggle with drug use, which is known to be a method of transmission for HIV [52]. They have disproportionately been to prison or jail, and many have been to a self-help group, suggesting that those could be effective venues for HIV outreach [53, 54]. They also struggle with drinking, indicating outreach may be possible at places where alcohol is served [55]. The other cluster is dominated by Hispanic members. This group does not struggle with drugs or alcohol to a large extent, and many have not been to prison or a self help group. They will have to be reached by other means, such as through family and social connections [56].

With respect to injected drug use, the potential for outreach reflected in one cluster’s combination of prostitution and prison has been a topic of research [57]. The combination of homelessness and injected drug use reflected in three clusters has also been studied [57], particularly in young MSM communities [58]. The final injected drug use cluster was characterized by troubles with alcohol, and this relationship has been studied widely [59, 60]. Each of these chosen clusters are made up mostly of older individuals, showing that intervention targeting areas other than young communities, particularly those dealing with homelessness, is important for reaching these groups.

Many of our results concerning homelessness are reflected in the literature. One of our clusters paired homelessness with sex workers [61], while two paired homelessness with the use of methamphetamine [62], heroin, or opiates [63]. Two other clusters were particularly affected by alcoholism, with one predominately recovering from alcohol use [64], while the other had over 20 alcoholic drinks in the last month [65]. Most of the individuals in these two clusters have been to self help group meetings such as Alcoholics Anonymous, showing that some participants benefit greatly from such treatment while others may require alternatives. The problem of homelessness impacts many areas of society and would benefit from further, more timely study, such as related to the problem of assuring the homeless receive COVID-19 vaccinations [66].

The last target variable was for individuals with some form of health insurance. One cluster identified subjects with HIV. Possession of insurance by those with HIV has been connected with lower viral rates and a smaller incidence of premature death [67]. A recent study found that, despite new government insurance options, “the USA trails other high-income countries in key HIV-specific metrics, including rates of viral suppression” [68]. A second cluster was distinguished by sex workers with many partners. Interventions like PrEP, which is expensive but covered by insurance [69], would be of interest to this group.

While the results obtained are relevant to public health, we also consider the network science implications of this work. In [16] the most successful graph inference methods were MB and glasso, both of which use lasso regression; however in [26] the kNN graph was found to be most useful. In the current work, kNN produced 3 of the 4 final graphs. KNN is a simple method with relatively low time complexity: it must compute distance between all pairs of nodes, which can be done in *O*(*n*^2^) time. Unlike regression-based methods, the time complexity is not dependent on the number of features. KNN is a versatile method in that its calculation of distance can be done in any number of ways. We used Euclidean distance, but cosine similarity, Manhattan distance, and other variations are possible. The correlation matrix used with CT is, in effect, also a distance matrix. The level of correlation can be thought of as a distance, with more correlated variables being closer to each other. The correlation thresholding creates something more like a geometric graph, choosing all neighbors within a given distance, as opposed to the *k* nearest. Interestingly, a correlation thresholding geometric graph was found to produce results superior to kNN in [28]. In the current work, CT was one of the most useful graphs, producing a successful clustering for HIV.

It is a well-known fact that feature selection produces superior results in clustering [37] by removing lower-performing features and helping to overcome the curse of dimensionality. That result is confirmed here. Three of the 4 top graphs were created using only 15 features.

Last, the clustering method results were mixed. The NBR-Clust framework was shown to produce useful clusters in both [16, 26]. That result has continued here, where NBR-Clust with integrity was responsible for 2 of the 4 top results. Computing integrity has potentially high time complexity. The process was sped up using a multi-processor approximation [70]. The purposefully low-complexity Leiden and Louvain algorithms also performed well, and were each responsible for one of the 4 top clusterings.

The design of the current study is subject to limitations with respect to both the methodology involved and the interpretation of its results. The SATHCAP survey was conducted over a decade before this study, and during that time HIV, drug use, homelessness, and insurance are problems that have been examined quite intensely. Knowing this, it is difficult to find new and unexpected communities, even with successful results. In addition, the world is quite remarkably different. For example, PrEP medications now help control the spread of HIV, which has changed behavior in ways not foreseen by the SATHCAP survey. In the United States, the purchasing of insurance has been made much easier (and in some cases required) by new laws.

A limitation of the methodology is that it is not able to determine an overall best clustering method, just the best given a particular set of clusters. The structure of most of our graphs meant that we ended up with what seems like a large number of clusters. For researchers looking for completely feature-disjoint clusters, it may be better to set an upper limit on their quantity. This is easily done with all the clustering methods. Last, our paper uses step-wise feature selection with logistic regression because of success in previous research. There are many feature selection methods available in the literature that may give better results and would be worth testing as future work.

## Conclusion

This paper presented a framework for applying graph inference techniques and clustering on health and medical data. We used the SATHCAP survey, which is typical of many medical datasets, suggesting that the methodology can be generalized to other datasets as well. The size of our data varied, ranging from 416 positive observations with the variable *HIV* to 1,982 positive observations with the variable *homeless*. Both kNN and correlation thresholding created graphs that gave relevant results, even when the target attributes were outside the original scope of the survey. NBR-Clust with integrity proved to be the most effective method for initial exploratory clustering, making up 2 of our 4 top partitions. In addition to finding associations that are reflected in the literature, our analysis demonstrated the usefulness of visualization of graph-based results. By identifying meaningful attributes associated with clustered groups, our methodology provided a basis for targeted intervention to help prevent HIV, to improve the lives of marginalized groups like IDUs and the homeless, and to show the importance of insurance in the mitigation of various health challenges.
